# Wearable Electrochemical Biosensors for Advanced Healthcare Monitoring

**DOI:** 10.1002/advs.202411433

**Published:** 2024-11-26

**Authors:** Haowei Duan, Shuhua Peng, Shuai He, Shi‐Yang Tang, Keisuke Goda, Chun H. Wang, Ming Li

**Affiliations:** ^1^ School of Mechanical and Manufacturing Engineering The University of New South Wales Sydney NSW 2052 Australia; ^2^ Department of Chemistry The University of Tokyo Tokyo 113‐0033 Japan; ^3^ Department of Bioengineering University of California Los Angeles California 90095 USA; ^4^ Institute of Technological Sciences Wuhan University Hubei 430072 China

**Keywords:** biomarkers, body fluids, electrochemical biosensors, healthcare monitoring, wearable electronics

## Abstract

Recent advancements in wearable electrochemical biosensors have opened new avenues for on‐body and continuous detection of biomarkers, enabling personalized, real‐time, and preventive healthcare. While glucose monitoring has set a precedent for wearable biosensors, the field is rapidly expanding to include a wider range of analytes crucial for disease diagnosis, treatment, and management. In this review, recent key innovations are examined in the design and manufacturing underpinning these biosensing platforms including biorecognition elements, signal transduction methods, electrode and substrate materials, and fabrication techniques. The applications of these biosensors are then highlighted in detecting a variety of biochemical markers, such as small molecules, hormones, drugs, and macromolecules, in biofluids including interstitial fluid, sweat, wound exudate, saliva, and tears. Additionally, the review also covers recent advances in wearable electrochemical biosensing platforms, such as multi‐sensory integration, closed‐loop control, and power supply. Furthermore, the challenges associated with critical issues are discussed, such as biocompatibility, biofouling, and sensor degradation, and the opportunities in materials science, nanotechnology, and artificial intelligence to overcome these limitations.

## Graphical abstract

Bioreceptor‐based wearable electrochemical sensors have opened new avenues for on‐body and continuous molecular detections, enabling personalized and preventive healthcare. This review summarises recent innovations and applications of those wearable biosensing systems in detecting a variety of biochemical markers, such as small molecules, hormones, drugs, and macromolecules, in biofluids including interstitial fluid, sweat, wound exudate, saliva, and tears.

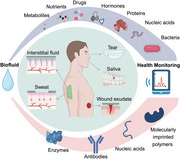

## 1 Introduction

Analytical devices for biomarker detection represent a rapidly advancing interdisciplinary field poised to revolutionize healthcare, including disease diagnosis, fitness management, and personalized medicine.^[^ ^1^, ^2^ ^]^ Wearable sensors, in particular, have become indispensable tools in modern healthcare due to their ability to continuously monitor vital physiological parameters. By enabling the in situ collection and real‐time detection of vital biomarkers directly from body fluids, wearable sensors can capture valuable and seamless insights into the health status of individuals without the need for frequent hospital visits.^[^ ^1^, ^3^ ^]^

Since the first modern biosensor developed by integrating glucose oxidase enzyme into oxygen electrode in 1962, the electrochemical biosensors, the most widely employed biosensing technology, have achieved considerable clinical and market success.^[^ ^4^, ^5^ ^]^ Among various signal transduction strategies, the electrochemical sensing approach offers ease of miniaturization and cost‐effectiveness compared to fluorescence and plasmonic methods.^[^ ^6^, ^7^, ^8^, ^9^, ^10^, ^11^, ^12^, ^13^ ^]^ Additionally, it can provide seamless and quantitative data with higher accuracy relative to colorimetric wearables,^[^ ^14^, ^15^ ^]^ making it particularly suitable for on‐body applications. For example, continuous glucose monitors (CGM), can accurately and continuously quantify glucose concentrations by converting enzyme‐catalyzed reactions into electrical signals in real‐time. These devices decentralize diabetes diagnostics and management from clinical laboratories, which often rely on intermittent testing and may be inaccessible in resource‐limited settings.^[^ ^16^ ^]^ Despite their successful application in commercial products for continuous and rapid measurements, enzymatic reactions are constrained by their ability to monitor only a limited number of targets directly.

The discovery of a broad range of health‐relevant biomarkers has spurred an increasing demand for sensors capable of targeting newly discovered biomarkers. Various biorecognition elements including antibodies,^[^ ^17^ ^]^ nucleic acids (e.g., aptamers),^[^ ^18^ ^]^ and molecularly imprinted polymers (MIPs),^[^ ^19^ ^]^ have been employed to selectively detect a wider array of specific biomarkers of great interest. For example, similar to the high target specificity provided by enzymes, wearable electrochemical biosensors utilizing affinity‐based bioreceptors demonstrate the potential for continuous tracking of drug molecules (e.g., antibiotics and anticancer agents) in living subjects, allowing for the evaluation of pharmacokinetic variability among individuals and the customizations of diagnostics and treatments to individual profiles.^[^ ^20^ ^]^ As new biorecognition elements are incorporated into body‐based platforms, the number of clinically relevant analytes that can be reliably detected is expected to expand, thereby enhancing the diagnostic potential of these biosensing devices.

**Figure 1:**
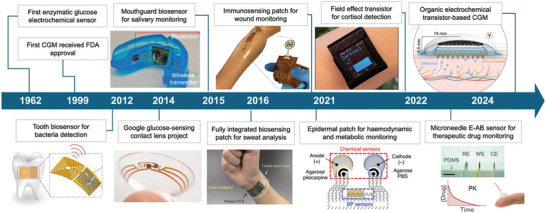
The evolution of bioreceptor‐based wearable electrochemical sensors. Tooth biosensor for bacteria detection. Reproduced with permission.^[^ ^30^ ^]^ Copyright 2012, Springer Nature; Google contact lens project for tear glucose detection. Reproduced with permission.^[^ ^31^, ^32^ ^]^ Copyright 2014, Google; A mouthguard‐based enzymatic sensor for detection of salivary uric acid. Reproduced with permission.^[^ ^33^ ^]^ Copyright 2015, Elsevier; Fully integrated biosensing patch for multiplex perspiration analysis. reproduced with permission. Copyright 2016, Springer Nature; Multiplexed immunosensing wound patch. Adapted under the terms of the CC‐BY license.^[^ ^34^ ^]^ Copyright 2021, The Authors; A hybrid patch combining acoustic and electrochemical biosensors. Reproduced with permission.^[^ ^35^ ^]^ Copyright 2021, Springer Nature; Aptamer field‐effect transistor (FET) wearable watch for cortisol detection. Reproduced under the terms of the CC‐BY license.^[^ ^36^ ^]^ Copyright 2022, the Authors; Wearable electrochemical aptamer‐based (E‐AB) microneedle sensor for therapeutic drug monitoring of tobramycin. Reproduced with permission.^[^ ^37^ ^]^ Copyright 2022, American Association for the Advancement of Science; A fully integrated organic electrochemical transistor (OECT)‐based CGM prototype. Reproduced under the terms of the CC‐BY license.^[^ ^38^ ^]^ Copyright 2024, the Authors.

With advancements in functional materials, manufacturing techniques, new device designs, and integration concepts, significant progress has been made in the field of wearable electrochemical biosensors. A brief timeline of the incorporation of key bioreceptors in wearable devices is summarized in **Figure** **1**. Over the past decades, these sensors have been extensively developed to enable real‐time, continuous measurement of various biomarkers directly on the body, such as metabolites, drugs, hormones, cytokines, and nucleic acids in blood surrogate biofluids. These on‐body sensing devices come in various forms, such as microneedles, wearable patches, tattoos, contact lenses, and mouthguard. The improvement in accuracy, effectiveness, and reliability have increased the commercial potential of these biosensing platforms. This progress marks a new era for wearable sensors, allowing for comprehensive and remote health monitoring, even in resource‐limited settings. Additionally, these advances are crucial for addressing healthcare access issues and reducing healthcare‐associated time and costs.

In this review, we summarize the latest developments in wearable electrochemical sensors, with a particular focus on the use of bioreceptors for real‐time and continuous biomolecular analysis. This focus differentiates our work from recent reviews on laboratory‐based and point‐of‐care electrochemical biosensors,^[^ ^21^, ^22^, ^23^, ^24^ ^]^ addressing the challenges and advancements involved in transitioning bioreceptor‐based sensors toward on‐body and long‐term measurements. Additionally, rather than focusing solely on wearables that utilize a single type of bioreceptors,^[^ ^25^, ^26^, ^27^, ^28^, ^29^ ^]^ this review explores both enzymes and various affinity‐based bioreceptors, offering comprehensive insights into emerging biorecognition elements. We first introduce various building blocks used to construct these biosensing platforms, and then highlight their achievements and advancements in on‐body applications across various biofluids, including interstitial fluid (ISF), sweat, wound exudate, saliva, and tears. We also discuss several recent advances, and the challenges associated with current sensing platforms, particularly for in *vivo* or on‐body measurements. Finally, potential future directions in for developing new wearable electrochemical biosensors are discussed, emphasizing recent advancements in materials science, nanotechnology, biochemistry, and artificial intelligence.

## 2 Construction of Wearable Electrochemical Biosensors

To construct a reliable biosensing interface for electrochemical wearables devices, it is crucial to carefully select suitable bioreceptors, signal transduction methods, electrode and substrate materials, and fabrication techniques. For example, while the intrinsic properties of a bioreceptor enable selective interaction and detection of target analytes, the immobilization of these bioreceptors requires careful consideration of electrode materials and interrogation techniques.^[^ ^39^ ^]^ Additionally, the stability and longevity of the bioreceptors, as well as the strength of the chemical bonds used for their immobilization, are key factors influencing analytical performance over extended periods and in the complex environment of human biofluids. Furthermore, the sensing system embedded in the human body must be biocompatible and flexible to ensure that it does not trigger adverse immune responses or pose safety concerns for wearers.

### 2.1 Bioreceptors

Bioreceptors, which are typically immobilized on the electrode surface for signal transduction, are considered as the cornerstone of wearable electrochemical biosensing platforms. These bioreceptors, such as enzymes, antibodies, aptamers, MIPs, ensure specific interaction between the target analyte and the electrochemical interface, effectively minimizing false signals.^[^ ^5^, ^40^ ^]^

Enzymes have pioneered the field of biosensing since the launch of the first electrochemical biosensor based on glucose oxidase in 1962.^[^ ^41^ ^]^ Most enzyme‐based sensors operate by coupling a redox event, generated upon the addition of a target, with signal transduction through direct or mediator‐bridged electron transfer to the electrode.^[^ ^42^ ^]^ A key advantage of enzyme‐based biosensors is that the catalytic turnover of the enzyme enables continuous signal transduction, making them particularly suitable for acquiring detailed temporal profiles of biomarkers.^[^ ^41^ ^]^ However, these sensors are generally effective for only a limited range of metabolites and drugs, such as glucose, lactase, and hydrogen peroxide.^[^ ^41^, ^43^ ^]^

Antibodies, as one of the most popular affinity‐based bioreceptors, are produced through the immune response to external stimuli. They have emerged as a novel prime for biosensing due to their high affinity and excellent selectivity.^[^ ^44^ ^]^ However, antibodies suffer from several limitations in biosensing applications, such as fragility to temperature and pH changes, low batch‐to‐batch consistency, and relatively slow binding kinetics.^[^ ^45^, ^46^ ^]^

Nucleic acids, particularly aptamers, offer promising alternatives to enzymes and antibodies in electrochemical biosensing. As a prime example, aptamers are short single‐stranded RNA or DNA oligos selected in vitro through Systematic Evolution Ligands Exponential Enrichment (SELEX) process. In addition to animal‐free production, ease of chemical modification, and adjustable binding affinity.^[^ ^47^, ^48^ ^]^ Aptamers undergo conformational changes by folding into unique structures upon specific binding to a variety of target analytes (e.g., small molecules, drugs, proteins, bacteria, and even whole cells).^[^ ^49^, ^50^ ^]^ Nevertheless, they are susceptible to the degradation of nucleases and sensitive to changes in environmental conditions, such as pH, temperature, and ionic concentrations of biological fluids.^[^ ^51^, ^52^ ^]^

MIPs, often referred to as “artificial antibodies”, have gained prominence in electrochemical biosensing since their inception by Wulff et al. in 1993.^[^ ^19^ ^]^ The synthesis of MIP involves a molecular imprinting process where functional monomers polymerize around a template molecule in the presence of cross‐linking agents. This forms a polymer matrix with cavities specifically shaped to match the template molecule. MIPs offer several advantages over biological antibodies, including enhanced chemical and thermal stability and cost‐effectiveness.^[^ ^53^ ^]^ However, the use of MIPs in wearable biosensors remains nascent, which is possibly hindered by challenges, such as limited selectivity toward structurally similar molecules, and reproducibility issues.^[^ ^54^, ^55^ ^]^

### 2.2 Signal Transduction

**Figure 2:**
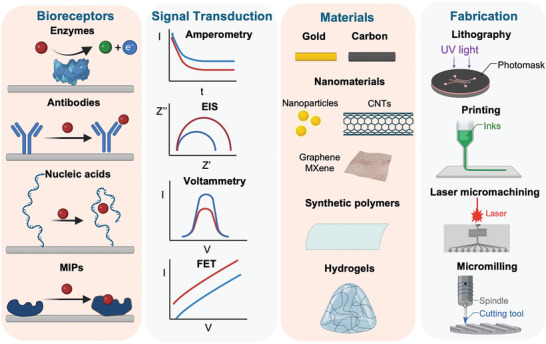
Schematic illustration of wearable electrochemical biosensors, highlighting their construction based on bioreceptors, signal transduction techniques, electrode and substrate materials, and fabrication approaches.

Signal transduction of target binding by a bioreceptor can be achieved through a range of electrochemical detection strategies (**Figure** **2**), such as amperometry, voltammetry, and impedimetric techniques, each with its own characteristics and strengths.

Amperometry involves the continuous measurement of current under a controlled potential from redox reactions, which is well suitable for transducing enzymatic reactions.^[^ ^56^ ^]^ The current density in a typical current versus time (I‐t) curve obtained from amperometric measurements can be directly correlated with the concentration of the target. While amperometry is also versatile for transducing enzyme modified antibodies or nucleic acids, the requirement for additional labels and multiple washing steps hinder its applications for rapid and continuous molecular measurements.^[^ ^57^ ^]^

Electrochemical impedance spectroscopy (EIS) is a label‐free method that quantifies target concentrations through detecting target‐induced variations in electrochemical interface properties without additional labeling of the bioreceptors.^[^ ^58^, ^59^ ^]^ EIS achieves this by directly investigating detail‐rich interfacial variations associated with bioreceptor‐analyte recognition events through non‐faradaic parameters (e.g., double‐layer capacitance and faradic impedance).^[^ ^59^, ^60^ ^]^ However, an inherent limitation of EIS interrogation is its low selectivity, which hinders its practical applications and effectiveness in in vivo measurements.

Voltammetry, such as differential pulse voltammetry and square wave voltammetry, is also widely used technique for wearable electrochemical biosensors. During voltammetric measurements, the resultant current changes under methodically altered potentials are recorded to monitor the faradaic response of electroactive moieties (e.g., methylene blue and ferrocene) conjugated to the bioreceptors or electroactive MIPs.^[^ ^61^ ^]^ As a source of background noise, the non‐faradaic current subject to the changes of electrolyte compositions and non‐target interferents, can be minimized by voltammetric methods, thereby enhancing the sensitivity of detection.

FET is another powerful label‐free technique for signal transduction and has been integrated into wearables.^[^ ^3^, ^62^, ^63^ ^]^ A FET consists of a gate channel (such as PEDOT: PSS,^[^ ^64^ ^]^ metal oxides^[^ ^36^ ^]^) where biorecognition elements are attached. Upon target binding, a variation in the electrical interfacial properties of the gate channel results in a shift in the drain‐source current, which is dependent on the analyte concentration. Additionally, the complete penetration of ions in the electrolyte during FET measurements, essential for semiconductor doping, generates large drain currents even with a small gate voltage. Such unique mechanism makes FET‐based biosensors excellent at amplifying signals, especially for detecting low‐abundance biomolecules.^[^ ^65^ ^]^

### 2.3 Materials

Materials used as both electrodes and substrates in wearable electrochemical biosensors must possess several essential characteristics. The materials that can be utilized as electrodes should have high conductivity, ease of manufacturing, a high surface area, and ideally, biocompatibility. To ensure effective contact between the human body and wearable bioelectronics, a soft, tissue‐like substrate is preferable, as it conforms well to the rugged and irregular surfaces of the body. This section summarizes the most popular materials with notable physiochemical properties and suitability for use as electrodes and substrates in wearable electrochemical sensing.

#### 2.3.1 Electrode Materials

Electrode, serving as an interface for the attachment of bioreceptors and the transduction of target recognition events, determines several key characteristics of biosensors used for on‐body measurements. These characteristics include analytical performance, interrogation scheme, and longevity during storage and operation. Given the wide range of electrode materials available, selecting the optimal candidature is crucial and should consider several unique factors when integrating bioreceptors with wearable platforms. For example, electrodes that come into direct contact with the human body must be biocompatible with excellent fouling resistance.^[^ ^66^ ^]^ Another important factor is the capability of the electrode to anchor biorecognition elements.^[^ ^67^, ^68^ ^]^ Various pathways, such as biotin‐avidin conjugation, thiol‐metal interaction, or amine‐carboxylic binding, can be used to attach biorecognition elements to different types of electrode surfaces.^[^ ^69^ ^]^

To date, carbon and gold materials have been extensively explored for constructing wearable electrochemical transducer device due to their high stability, excellent electrochemical properties, wide operational potential window, cost‐effectiveness, and ease of surface modification.^[^ ^70^, ^71^, ^72^, ^73^ ^]^ For example, immobilizing a self‐assembled monolayer on a gold surface or carboxylic carbon has become a predominant strategy for anchoring biorecognition elements. This approach is favored for its simplicity of the involved one‐pot conjugation and the ability of the monolayer to be backfilled, which helps prevent nonspecific absorption and allows for prolonged on‐body monitoring.^[^ ^67^ ^]^

Additionally, various functional nanomaterials, including metallic nanoparticles,^[^ ^74^ ^]^ carbon nanotubes, MXene,^[^ ^75^ ^]^ metal‐organic framework, graphene,^[^ ^76^ ^]^ nanoporous gold,^[^ ^77^ ^]^ and conducting polymers have been incorporated into the working electrodes of electrochemical sensors. These materials enhance the electroactive area, electron transfer efficiency, and bioreceptor loading ability, collectively improving the analytical performance of wearable sensing devices. However, safety concerns regarding foreign‐body responses and inflammation from nanomaterial‐modified sensing devices should be carefully evaluated and validated on human subjects.

#### 2.3.2 Substrate Materials

To integrate devices for biosensing applications, synthetic polymers and hydrogels are commonly used materials for creating patterns to collect and transport body fluids, and to embed electrodes.^[^ ^78^ ^]^ Among the flexible substrates for printed electronics, synthetic polymers are the most used substrate for on‐body sensing applications, due to their flexibility, biocompatibility, and ease of mass scale production. Several inert and biocompatible elastomers,^[^ ^78^ ^]^ including silicone, polyethylene terephthalate (PET), polyimide (PI), polydimethylsiloxane (PDMS), and poly(styrene–butadiene–styrene), are particularly suitable for long‐term and continuous monitoring because they are skin safe options.^[^ ^79^ ^]^ By matching their mechanical properties (e.g., elasticity and stretchability) with those of human tissues, the flexible nature of polymeric substrates enables tight and conformal contact with biological subjects.^[^ ^80^ ^]^ Moreover, the properties of polymeric substrates can be further modified through physical (e.g., hydrophilicity via oxygen plasma) and chemical functionalization.^[^ ^1^ ^]^

Alternatively, hydrogels, including poly(vinyl alcohol) (PVA), alginate, and gelatine, represent another promising category of substrate materials for biosensing applications.^[^ ^81^ ^]^ Compared to other polymer systems, hydrogels tend to be more expensive to synthesize, which restricts their use to specialty domains. Currently, hydrogels are primarily employed as matrices to enhance sample collection and transport, as electrode support materials for electrode coatings to prevent biofouling, and as drug loading reservoirs for on‐demand delivery. For example, hydrogels with natural porosity and hydrophilicity can effectively permeate and transport liquids, facilitating biofluid sampling and reducing lag time during biosensing. Compared to synthetic polymers, hydrogels are more biocompatible, marking them well‐suited for on‐body applications involving direct contact with the skin, bodily fluids, and wound areas.^[^ ^82^, ^83^ ^]^ Despite these unique features, the use of hydrogels as the substrate materials in wearable biosensors remains relatively limited, possibly because of the mechanical mismatch between hydrogels and sensing electrodes. Specifically, electrode materials often have lower stretchability compared to hydrogels,^[^ ^79^ ^]^ which can result in disconnections as well as inconsistent performance during long‐term exposure to biofluids.^[^ ^84^ ^]^

### 2.4 Fabrication

The fabrication of electrochemical sensors involves various techniques and materials to achieve the desired design, functionality, and performance. There are several fabrication methods available for producing sensing substrates, including lithography, printing, and additive techniques such as 3D printing, laser micromachining, and micromiling.^[^ ^1^, ^80^, ^85^, ^86^ ^]^

Lithography is popular for fabricating wearable electrochemical devices, due to their excellent pattern resolution, high uniformity, and reproducibility. Different types of lithograph techniques, such as electron beam lithography, iron beam lithography, photolithography have been adopted to transfer a 2D pattern of electrodes onto the substrates. For example, photolithography is a light‐based microfabrication technique commonly used to create high‐resolution patterns and complex structures with feature sizes down to the nanoscale.^[^ ^87^, ^88^ ^]^ However, the complicated processing and expensive equipment needed for these conventional cleanroom facility based methods can be obstacles.^[^ ^89^ ^]^

Printing has been adopted to efficiently pattern electrode circuits on deformable substrates.^[^ ^90^ ^]^ For example, the emergence of screen printing and additive fabrication (i.e., 3D printing) has generated great interest as a unique versatile tool for rapidly producing prototypes over a wide range of material choices,^[^ ^86^ ^]^ including nanomaterials, polymers, and hydrogel.^[^ ^8^, ^91^ ^]^ These simple and cost‐effective methods are particularly advantageous for customizing wearable sensing prototypes for healthcare surveillance, given the demand for integrating diverse modalities into compact wearable devices.

Laser micromachining stands as a pivotal technology in the manufacturing of wearable sensor electronics, leveraging focused laser beams to sculpt and structure materials (e.g., gold, platinum, silver, graphene,^[^ ^92^ ^]^ and polymeric substrates) with excellent precision.^[^ ^91^, ^93^ ^]^ Particularly when working with the delicate circuits and thin films of the sensor devices, laser micromachining minimizes thermal damage, while enabling the creation of intricate microscale features and patterns essential for the high functionality of wearable sensors.^[^ ^94^ ^]^

Micromilling is another widely used fabrication method for creating wearable sensors from a starting stock piece. This technique involves using fine, precision‐engineered rotating cutting tools to rapidly carve microscale features directly into plastic substrates.^[^ ^95^ ^]^ In the context of wearable sensor electronics, micromilling is also versatile, capable of handling a variety of plastic materials (e.g., polystyrene, polymethyl methacrylate) to meet specific design requirements, making it ideal for prototyping and small batch production.^[^ ^96^ ^]^

## 3 Epidermal Wearable Sensors

Wearable electrochemical biosensors have been developed in diverse platforms depending on their application and the body fluids being tested. Epidermal wearable biosensors, which comfortably conform to the human skin, are used for monitoring biomarkers in human interstitial fluid, sweat, and wound exudate. Devices, such as patches, smartwatches, wristband, and skin‐worn biosensors have been commonly designed for monitoring of biomarkers in epidermal biofluids, including small molecules (e.g., metabolites and nutrients), hormones, drugs, and macromolecules (e.g., proteins and nucleic acids). Notably, ionic species (e.g., sodium, potassium, calcium, ammonium, and chloride) and biofluidic pH are also crucial biomarkers for monitoring electrolyte balance, hydration status, and diseases (e.g., bacterial infections and cystic fibrosis).^[^ ^86^, ^97^ ^]^ Since this field is relatively mature, and the recognition elements used (i.e., ionophores for different ions,^[^ ^98^, ^99^, ^100^, ^101^ ^]^ and conducting polymers for pH measurements) are not typically classified as bioreceptors,^[^ ^102^, ^103^ ^]^ monitoring electrolyte conditions is not included within the scope of this review.

### 3.1 Wearable ISF Sensors

Selecting a suitable biological fluid is critical for accurately deriving clinically relevant biomarkers for on‐body applications and determining the modalities of wearable sensors. A promising biofluid for sensing applications should contain diverse biomarkers of interest, reflect clinically relevant concentration, and ideally be sampleable and collectable noninvasively. Among various biofluids, ISF stands out because it surrounds cells and tissues and exchanges substrates with blood capillaries. It can be accessed and collected more easily than blood, which typically involves pain or clotting issues.^[^ ^104^ ^]^ Another driving force behind the growing interest in ISF‐based diagnostics is its expanded clinical relevance and biomarker diversity compared to other accessible biofluids (such as saliva, sweat, and tears). Since ISF contains almost all the analytes present in the blood, diagnostic assessments in ISF only require evaluating the correlation of targets in ISF with those in blood, rather than simply determining the presence of the analyte.^[^ ^105^ ^]^ This compositional similarity to blood makes in situ analysis of ISF a promising alternative to blood‐based clinical measurements, enabling minimal‐invasive monitoring of physiological status and disease management.

#### 3.1.1 Techniques for ISF Sampling

**Figure 3:**
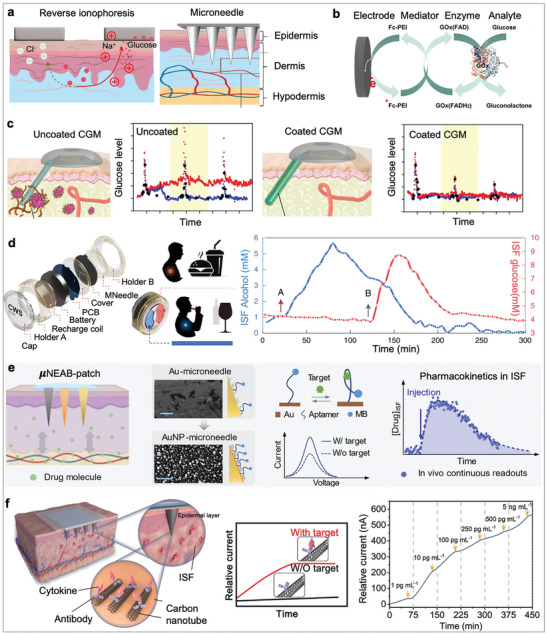
Interstitial fluid‐based wearable electrochemical biosensors. a) Schematic illustrations of RI‐ and microneedle‐based approaches for ISF sampling. Left: A typical RI involves extracting ISF from vessels and transporting molecules to cathode electrode via a current driven process. Right: Schematic of a microneedle patch embedded under the skin for continuous glucose monitoring. Reproduced under the terms of the CC‐BY license.^[^ ^97^ ^]^ Copyright 2024, Springer Nature. b) Sensing principle of the CGM, which uses a mediator for rapid electron transfer between glucose oxidase and the electrode. Reproduced with permission.^[^ ^116^ ^]^ Copyright 2023, American Chemical Society. c) Uncoated sensors induce an inflammatory immune cascade while zwitterionic polymer‐coated sensors overcome the hostile i*n vivo* environment. Adapted with permission.^[^ ^119^ ^]^ Copyright 2018, Springer Nature. d) An integrated MN patch for multiplexed metabolite detection. Left: Overview of the wearable MN patch, which integrated 9 different components into a single compact electronic device. Right: Continuous on‐body performance of the wearable MN patch after consumption of wine and meal. The plot represents alcohol and glucose concentrations measured in ISF over 5 h. Reproduced with permission.^[^ ^121^ ^]^ Copyright 2022, Springer Nature. e) Wearable E‐AB MN sensor can continuously track different levels of tobramycin. AuNP‐engineered microneedles render high aptamer coverage and efficient transduction of voltammetric signals upon target binding. Reproduced with permission.^[^ ^37^ ^]^ Copyright 2022, American Association for the Advancement of Science. f) Antibody‐modified MN sensor for in vivo monitoring of cytokine levels. Reproduced with permission.^[^ ^127^ ^]^ Copyright 2023, Wiley‐VCH GmbH.

To date, sampling and measurement of ISF have been achieved either by withdrawing (e.g., wick extraction, suction blister, reverse iontophoresis) or by using indwelling electrodes (e.g., microneedles) that remain under the skin surface and are immersed in ISF. For withdrawal techniques, inserting absorbent materials such as nylon or hydrogel into skin interstices is a conventional technique to extract ISF. However, this approach can only collect small volumes of ISF (e.g., a patch with 100 hydrogel absorbent can collect only 24 nanoliters of ISF per hour).^[^ ^106^ ^]^ Additionally, the protein dilution effect and slow concentration equilibrium of absorbent materials further hinder their application in biosensing. Reverse iontophoresis (RI) is another method for ISF collection, which applies a voltage to produce sufficient and controllable ISF samples (**Figure** **3**a, **left)**. For instance, the FDA‐approved wearable wrist sensor (GlucoWatch Biographer) first used RI for continuous, non‐invasive glucose monitoring for up to 12 h, with a sampling rate of every ten minutes. However, the approval was withdrawn later due to issues, such as a long warm‐up period (2‐3 h), severe skin irritation, and the need for calibration using an invasive glucometer. Another concern with RI approach is the concentration distortion of the analytes during extraction, as the composition of collected ISF can differ significantly from its actual status.^[^ ^107^ ^]^

Alternatively, microneedle (MN)‐based ISF extraction and diagnostics (Figure 3a, right) have shown significant potential. Microneedles, typically less than 1000 micrometers in length,^[^ ^108^ ^]^ offer a straightforward approach for ISF extraction compared to the previously mentioned withdrawal methods. Depending on their materials and structures, common microneedle types include solid, coated, hollow, gel, and porous forms, with porous and hollow microneedles capable of extracting ISF at rates of 4 to 16 microliters per minute. However, it remains uncertain whether ISF samples extracted using these needles can be reliably obtained without altering the concentrations of analytes. For example, wearable devices that use dermal compression or strong suction may disrupt the pressure balance between capillaries, ISF, and lymphatic capillaries, potentially altering analyte concentrations.^[^ ^109^, ^110^ ^]^

#### 3.1.2 Wearable ISF Sensor for Small Molecule Detection

Endogenous small molecules (with a molecular weight of hundreds of Daltons) in ISF, e.g., nutrition and metabolites, often exhibit strong concentration correlations with those in blood. Monitoring these molecules through wearable ISF sampling devices holds great promise to replace blood‐dominated biomarker detections. Among numerous analytes, monitoring glucose fluctuations in ISF is particularly significant owing to its relevance in managing diabetes mellitus and reducing the risk of hyperglycaemia and hypoglycaemia.^[^ ^111^, ^112^, ^113^, ^114^ ^]^ Ealy advancements include a MN array functionalized through electropolymerized glutamate oxidase (GluOx) and glucose oxidase (GOx) embedded within poly(o‐phenylenediamine) (PPD) thin films.^[^ ^115^ ^]^ This design, which combines hollow and solid microneedles, allows for direct transdermal biosensing of glutamate and glucose without fluid extraction. It demonstrates high‐fidelity measurements of these substances in undiluted human serum, with detection limits of 10 µM and 0.1 mM, respectively. Such enzymatic sensing mechanism (Figure 3b), coupled with printed circuit boards (PCBs) and wireless transmission modules, has been commercialized in several continuous glucose monitors.^[^ ^116^ ^]^ For instance, the FreeStyle Libre CGM uses a spring‐loaded inserter (with a cross‐sectional area of 0.3 mm^2^ and a length of 8.5 mm) to apply the sensor patch to the skin. The deployed electrode is positioned ≈5 mm below the skin surface, providing continuous glucose readings over two weeks.

Unfortunately, the insertion of wearable MN is always accompanied with foreign body responses (FBR), including inflammation and fibrosis due to the wound healing process.^[^ ^117^, ^118^ ^]^ This severe host response can degrade analytical accuracy and increase signal noise, posing a major obstacle to the continuous and long‐term operation of wearable microneedle sensors. To mitigate FBR, a zwitterionic polymer (Figure 3c) selected through combinatorial chemistry was applied to the inner electrode of a commercial CGM (Medtronic MiniMed) through dopamine‐mediated conjugation of methacryloyloxyethyl phosphorylcholine.^[^ ^119^ ^]^ This coating effectively inhibits non‐specific biomolecule absorption due to the hydration layer on the zwitterionic polymer, reducing biofouling and capsular formation. Histological studies and gene‐expression profiling revealed that this coating abrogated or completely suppressed various cytokines and immune markers associated with FBR. In vivo evaluations in mice and non‐human primates demonstrated that the zwitterionic‐coated sensors showed significantly reduced signal fluctuation and maintained higher analytical consistency compared to uncoated sensors.

To address the discomfort associated with wearable MN patch, a completely non‐invasive RI approach has been developed for ISF detection. This non‐invasive skin‐like biosensor system integrates ultrathin, flexible biosensors with paper battery‐powered electrochemical twin channels (ETCs) for continuous intravascular blood glucose monitoring.^[^ ^120^ ^]^ The subcutaneous ETCs drive intravascular blood glucose out of vessels and transport it to the skin surface, where the biosensor measures it with high sensitivity (130.4 mA per mM). In vivo clinical trials demonstrated a high correlation (*r* > 0.9) between non‐invasive measurements and clinically measured blood glucose levels, showing significant promise for clinical‐grade glucose monitoring and advanced diabetes management. By replacing GOx immobilized within the sensing layer, wearable MN electrochemical sensors can also detect a variety of other endogenous molecules with high levels of integrity (Figure 3d).^[^ ^121^ ^]^ Examples include urease for urea,^[^ ^122^ ^]^ cholesterol oxidase for cholesterol,^[^ ^123^ ^]^ β‐hydroxybutyrate dehydrogenase for β‐hydroxybutyrate,^[^ ^124^ ^]^ ascorbate oxidase for vitamin C, and anti‐lactate aptamer^[^ ^125^ ^]^ or lactase oxidase for lactate.^[^ ^121^, ^126^ ^]^

#### 3.1.3 Wearable ISF Sensor for Drug Monitoring

In clinical practice, many drugs have a narrow therapeutic window, meaning that the administrated dose may be either insufficient to achieve the desired therapeutic outcome, or too high to tolerate without causing side effects. Therapeutic drug monitoring (TDM) involves measuring drug levels in a patient's blood at designated intervals to optimize drug dosage, ensure therapeutic efficacy, and minimize toxicity. This practice is essential for customizing the dosing of various drugs with interpatient variability in pharmacokinetics (PK), including anticonvulsants (e.g., phenytoin and valproate), antibiotics (e.g., vancomycin and aminoglycosides), immunosuppressants (e.g., cyclosporine and tacrolimus), and antipsychotics (e.g., lithium and clozapine). Traditionally, TDM is conducted through intermittent blood sampling followed by remote laboratory analysis, which is invasive and often leads to delayed dose adjustments, and impaired patient compliance. Additionally, TDM is not ubiquitously available due to its costs and the need for trained personnel and non‐standardized protocols.^[^ ^128^ ^]^

Alternatively, wearable ISF biosensors offer a promising approach to streamlining the TDM process by enabling minimally invasive and continuous drug monitoring. Unlike plasma‐based methods that typically measure the concentrations of drugs bound to proteins, ISF probing can detect drugs that are not protein‐bound, providing a more accurate reflection of active drug levels in the body.^[^ ^129^, ^130^ ^]^ Immediate access to drug levels allows patients and clinicians to be timely informed about pharmacokinetics, which can further improve adherence to prescribed regimens and overall health outcomes.

Early ISF drug monitoring relied on enzymatic bioreceptors, achieving continuous detection of antibiotics and nerve agents (e.g., opioid, organophosphate,^[^ ^131^, ^132^ ^]^ and levodopa).^[^ ^133^ ^]^ In 2019, Rawson et al. reported the first‐in‐human evaluation of an enzymatic ISF microneedle sensor for real‐time, in vivo tracking of the antibiotic phenoxymethylpenicillin. This proof‐of‐concept demonstration of a wearable MN sensor for TDM featured an array of solid microneedle electrodes coated with a hydrogel layer containing β‐lactamase enzyme and three iridium oxide‐modified electrodes for measuring pH changes.^[^ ^134^ ^]^ As the penicillin drug diffused into the hydrogel layer from the extracellular fluid, it was hydrolyzed by β‐lactamase to produce catalytic substrates including penicilloate and protons. The protons (H^+^) generation led to pH changes in the extracellular fluid, allowing for penicillin levels to be measured with accuracy comparable to microdialysis and patient serum measurements. Another example involves an enzymatic MN sensor designed by Goud et al.^[^ ^133^ ^]^ for highly selective detection of the antiparkinsonian drug L‐Dopa. This sensor, which uses chronoamperometric and SWV at tyrosinase‐packed and unmodified electrodes, respectively, demonstrates high selectivity even in the presence of potential interfering substances, including ascorbic acid, uric acid, tyrosine, carbidopa, and theophylline.

Recently, E‐AB techniques have been introduced into MN patches to expand the range of detectable drugs in ISF. Wang's Group first showcased the potential of E‐AB microneedle sensors for continuous tracking of several antibiotics, including tobramycin, doxorubicin, and vancomycin.^[^ ^135^ ^]^ Lin et al.^[^ ^37^ ^]^ reported a low‐cost fabrication method for transforming clinical‐grade needles into in vivo biosensing devices through the electrochemical deposition of gold nanoparticles (AuNPs) on acupuncture needles (Figure 3e). Leveraging the needle's porous structure, mechanical robustness, and improved surface area for efficient ISF extraction and signal retrieval, the wearable MN E‐AB patch achieves high signal‐to‐noise ratios and stable long‐term measurements of drug dynamics in the peripheral compartment (i.e., ISF). This allows for real‐time and continuous ISF pharmacokinetic profiling of tobramycin and vancomycin, providing valuable insights into total drug exposure and enabling precise adjustments within the therapeutic window.

Another innovative advancement in MN biosensing involves addressing sensor retraction and enhancing signal stability by placement. Traditionally, most MN biosensors are secured with medical glue, which can be incompatible with the mechanically soft and curved skin, leading to sensor retraction and diminished signal over time. A notable placement scheme involves using metallic plates subcutaneously to stabilize magnet‐containing E‐AB sensing patches during pharmacokinetic measurements. These aptamer‐based MN arrays, fabricated by stereolithography (SLA) for high resolution (≈10 µm) and rapid prototyping,^[^ ^136^ ^]^ are assembled with a ring‐shaped permanent magnet. Attracted by the surgically implanted metallic plate, the MN patches are affixed to the skin of rodents under magnetic force. This allows for continuous detection of tobramycin with enhanced signal retention.

#### 3.1.4 Wearable ISF Sensors for Macromolecular Detection

Nearly 90% of macromolecules circulating in the bloodstream are also presented in ISF, including crucial biomarkers such as cytokines, antibodies, and nucleic acids.^[^ ^137^, ^138^ ^]^ However, progress in wearable MN biosensing of macromolecules has been limited until the integration of wearable MN interfaces with affinity‐based bioreceptors, including antibodies, aptamers, and MIPs.

Cytokines, which are proteins involved in mediating immune responses and haematopoiesis, are closely linked to numerous physiological disorders and pathological conditions, such as trauma, sepsis, cancers, rheumatic diseases, and inflammatory bowel diseases. Along with increasing recognition of the clinical significance of cytokines in ISF, there has been growing interest in developing wearable MN sensors for tracking vital cytokines in ISF, such as interleukins‐6 (IL‐6),^[^ ^139^ ^]^ IL‐8,^[^ ^140^ ^]^ vascular endothelial growth factor,^[^ ^141^ ^]^ and epidermal growth factor receptor.^[^ ^142^ ^]^ However, most of these works rely on a two‐step process involving in vivo capture followed by in vitro analysis, which is label intensive and not suitable for continuous interrogation, making them less ideal for real‐time diagnosis.

A notable advancement (Figure 3f) is the first continuous MN immunosensor designed for capturing and quantifying cytokines (e.g., IL‐6) directly in ISF.^[^ ^127^ ^]^ This microneedle patch, functionalized with carbon nanotubes, allows real‐time measurements of IL‐6 concentrations through reductions in current intensities caused by steric hindrance effect upon cytokine binding. In vitro experiments showed a concentration‐dependent current intensity for IL‐6 ranging from 0 to 5000 pg mL^−1^, with a detection limit of 0.54 pg mL^−1^. Further in vivo evaluations demonstrated accuracy comparable to ELISA and the ability to provide early warnings of cytokine storms in a rat model of sepsis.

With the aid of nucleic acid receptors, wearable MN biosensing has also expanded to detect nucleic acids circulating in ISF for diagnosing viral infections. For example, Yang et al.^[^ ^143^ ^]^ developed a flexible MN patch for the on‐body electrochemical sensing of Epstein‐Barr virus cell‐free DNA (EBV cfDNA), a biomarker associated with nasopharyngeal carcinoma.^[^ ^144^ ^]^ The hydrogel MN patch samples ISF DNA, which is then hybridized with complementary single‐stranded oligonucleotide probes embedded in the hydrogel matrix, followed by amplification through recombinase polymerase amplification (RPA) and electrochemical testing. Although in vivo assessment achieved a detection limit of 370 000 copies mL^−1^, clinically relevant levels of EBV cfDNA can be as low as 134 copies mL^−1^,^[^ ^145^ ^]^ indicating a need for further improvement in sensitivity. For amplification‐free, in vivo monitoring of nucleic acids, the same group explored novel and universal nucleic acid sensing mechanisms, including CRISPR‐Cas9 and prokaryotic argonaut technology using MN electrochemical sensors.^[^ ^146^, ^147^ ^]^ In the latter case, they demonstrated the capability for real‐time detection of cell‐free DNA and RNA with femtomolar sensitivity over 14 days, paving the way for advanced on‐body nucleic acid detection.

Owing to their excellent compositional similarity and close analyte correlation with blood, wearable ISF sensors have demonstrated considerable capability for providing accurate biomarker profiling beyond glucose. However, several challenges remain. For example, the use of microneedle based platforms to disrupt the epidermis always accompanies with inflammation, which can break down capillary barriers and allow unimpeded diffusion of proteins and other large molecules,^[^ ^148^ ^]^ thereby affecting the accuracy of ISF measurements. In addition, the partitioning of macromolecules from blood involves more complex transport pathways (e.g., the extra filtration effect of the mesh‐like extracellular matrix within the interstitial space).^[^ ^149^ ^]^ As a result, solutes with molecular weights larger than 70 kDa (e.g., immunoglobulin antibodies) typically exist at lower picomolar concentrations, with their levels inversely related to the logarithm of their molecular weight.^[^ ^107^ ^]^ Therefore, fundamental research regarding their partitioning pathway, along with significant improvements in the limit of detection for bioreceptors, is needed to achieve robust clinical performance and commercial success.

### 3.2 Wearable Sweat Sensors

#### 3.2.1 Sweat Sampling Methods

While sweat is considered as one of the most obtainable non‐invasive biofluids, with more than 100 skin glands per cm^2^, its effective sampling requires sweat to be excreted onto the outer skin surface for biosensing analysis. Thus, the effectiveness of sweat generation and collection methods is pivotal to the success of wearable sweat sensors.^[^ ^150^, ^151^, ^152^ ^]^

**Figure 4:**
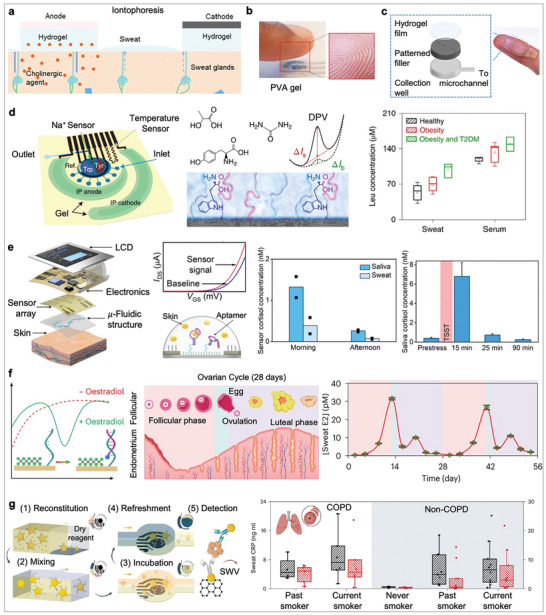
Sweat‐based wearable electrochemical biosensors. a) Schematics of iontophoresis‐based sweat stimulation. Reproduced with permission.^[^ ^86^ ^]^ Copyright 2023, American Chemical Society. b) PVA hydrogel‐based absorbent for sweat collection. Reproduced with permission.^[^ ^153^ ^]^ Copyright 2021, American Chemical Society. c) Microfluidic‐based methods for sweat collection. Reproduced under the terms of the CC‐BY license.^[^ ^155^ ^]^ Copyright 2021, The Authors. d) A MIP‐based sweat sensor for continuous profiling of various amino acids and nutrients. The leucine/BCAAs ratio in sweat exhibits a good correlation with blood levels and is elevated in individuals with diabetes and obesity. Reproduced with permission.^[^ ^156^ ^]^ Copyright 2022, Springer Nature. e) An aptamer‐based FET smartwatch for detecting sweat cortisol. During standard Trier Social Stress Test (TSST), the cortisol sensor monitors stress levels in participants. Reproduced under the terms of the CC‐BY license.^[^ ^36^ ^]^ Copyright 2022, The Authors. f) Monitoring of female hormone in sweat using an aptamer‐based wearable patch. This patch employs a target‐induced aptamer displacement strategy for in situ quantification, allowing tracking of hormonal fluctuations in sweat over the menstrual cycle. Reproduced with permission.^[^ ^157^ ^]^ Copyright 2023, Springer Nature. g) Monitoring of C‐reactive protein in sweat using an immunosensing wearable patch. This microfluidic system performs fully automatic sweat sampling, reagent routing, and target detection. The wearable patch can differentiate CRP levels in sweat among participants with chronic obstructive pulmonary disease and varying smoking statuses. Reproduced with permission.^[^ ^158^ ^]^ Copyright 2023, Springer Nature.

Sweat generation: Sweat can be generated through various pathways, including thermal or exercise stimulation, natural secretion, and iontophoresis. For example, exercise induction, which utilizes the body's natural response to physical activity (e.g., stationary biking, running), can provide controlled sweat secretion. However, variability in sweat rate and composition with respect to different exercise intensities poses challenges for consistent analysis. Alternatively, natural sweat secretion during daily activities has gained increasing attention, as it does not require external stimulation. Over the past decade, iontophoresis has become an emerging approach for stimulating sweat through chemical stimulation. As shown in **Figure** **4**a, iontophoresis utilizes a mild current flux generated between a pair of skin‐mounted electrodes, through which a cholinergic agonist (e.g., acetylcholine, methacholine, and pilocarpine) is released from the anode hydrogel and then delivered into the sweat glands. This method provides a controlled and localized sweat induction directly under the iontophoresis gel placement.

Sweat Collection: Once sweat is induced, its efficient harvesting and transport to the sensing area is critical. From an engineering perspective, sweat collection methods fall into two categories: absorbent‐based sampling and microfluidic driven sampling. Early methods of sweat collection involved absorbent materials, such as gauze pads, filter papers, and hydrogel patches (Figure 4b).^[^ ^153^, ^154^ ^]^ These materials are easily assessable and cost‐effective for single‐use purposes and point‐of‐care testing. However, absorbent‐based methods face challenges, such as saturation, contamination, and variability in absorption rates, which can affect the accuracy and reliability of subsequent analyses. Additionally, the potential for sweat sample saturation requires timely collection to prevent evaporation or contamination, making these methods suitable for applications in continuous monitoring scenarios. Microfluidic devices (Figure 4c) represent a significant advancement in sweat sampling by utilizing pressure‐driven microchannels to direct sweat into small reservoirs.^[^ ^155^ ^]^ These devices enable precise and continuous sweat collection, minimizing contamination and facilitating real‐time monitoring. Microfluidic systems can be integrated into wearable sensors, enhancing their practicality and effectiveness. However, the design and material properties of microchannels are crucial for efficient sweat transport and collection, and any flaws can lead to inaccuracies. Moreover, the cost and complexity of manufacturing microfluidic devices may limit their widespread adoption compared to simpler absorbent‐based methods.^[^ ^8^ ^]^

#### 3.2.2 Wearable Sweat Sensors for Small Molecular Detection

To date, wearable sweat sensors have successfully detected numerous small metabolic molecules, including key metabolites such as glucose, lactate, and uric acid. Among these, the sweat glucose sensor has attracted widespread and contentious interest as a completely non‐invasive alternative to blood and ISF biofluid measurements. In human subjects, glucose molecules enter sweat via eccrine glands, which exemplifies partitioning pathways and the effects of dilution on concentration.^[^ ^150^, ^151^, ^152^ ^]^ Specifically, while the paracellular pathway is relatively large (greater than 10 nanometers between cells), it involves high filtration due to tight junctions composed of various proteins. This leads to the dilution of most analytes in sweat, including glucose, resulting in sweat glucose levels (0.06 to 0.2 mM) that constitute only ≈1% of the glucose concentration found in ISF and plasma (3.3 to 17.3 mM). This significant dilution poses a major challenge to the sensitivity of the sweat glucose sensor.

A notable advancement in sweat glucose monitoring is the development of a wristband sweat sensor, which represents the first fully integrated platform capable of simultaneously detecting multiple physiological conditions of sweat, such as levels of metabolite and electrolytes, and skin temperature).^[^ ^150^ ^]^ This platform bridges the gap between data transduction, in situ signal processing, and wireless communication within a conformal PCB, allowing real‐time monitoring of fitness parameters during prolonged excise. Moreover, Wang's group reported an iontophoresis‐based tattoo platform, where iontophoresis electrodes and glucose sensing electrodes were fabricated through screen printing.^[^ ^159^ ^]^ To address skin irritation issues associated with iontophoresis devices like the GlucoWatch, the tattoo‐based glucose sensor limits the interrogation frequency and reduces the applied iontophoresis current to minimize discomfort. In addition, the flexible nature of the tattoo substrate conforms well to the body's contours, ensuring strong skin adhesion without impeding movement. The performance of this tattoo sensor was evaluated in healthy human subjects, demonstrating its ability to track sweat glucose variations over 20 h. Similar capabilities have also been demonstrated on a carbon nanotube modified woven fabric platform,^[^ ^160^ ^]^ which exhibits high resistance to deformation and endures mechanical stresses during body motion.

Lactate in sweat is another metabolic byproduct produced during intense physical activity. The level of lactate in sweat serves as an indicator of exercise efficiency and muscle fatigue without the need for invasive blood sampling. Various sweat biosensors for lactate detection have been developed, including lactate oxidase enzyme‐modified temporary tattoos,^[^ ^161^ ^]^ FET‐based electronic,^[^ ^162^ ^]^ wearable wristbands,^[^ ^150^ ^]^ and MIP grafted graphene‐printed electrodes.^[^ ^163^, ^164^ ^]^ These sensors profile lactate levels in human subjects, indicating exertion and exercise intensity. Although these studies consistently report positive correlations between sweat lactate and exercise intensity, it is important to note that sweat lactate levels do not directly reflect blood lactate levels. This discrepancy may arise from several factors, such as the unclear mechanism of lactate transport from plasma to sweat, lactate generation by epidermis sweat glands, and the dilution effect due to variable sweat rates.^[^ ^165^, ^166^, ^167^ ^]^

Sweat sensing of other metabolites indicating renal function, such as uric acid,^[^ ^168^, ^169^ ^]^ urea,^[^ ^169^ ^]^ and creatinine^[^ ^170^ ^]^ has also been achieved. For example, Han et al. reported an electronic skin comprising a piezoelectric power supply unit and an enzyme/ZnO nanoarrays matrix for detecting metabolic compounds, such as uric acid and urea.^[^ ^169^ ^]^ The sensing capability is attributed to the coupling effect between enzyme reactions on the electrode surface and the piezoelectric properties of ZnO nanowires, generating multimodal signals for uric acid and urea detection. However, the partitioning mechanism of these metabolites, like lactate, has not been explored in detail.

Moreover, monitoring of nutrients that are not enzyme reactive, including amino acids, has been achieved using novel bioreceptors.^[^ ^171^, ^172^, ^173^ ^]^ For example, Gao's group developed a MIP‐based sweat sensor (Figure 4d) for continuously quantifying sweat leucine and branched‐chain amino acids (BCAAs, such as isoleucine and valine),^[^ ^156^ ^]^ which are associated with obesity, insulin resistance, cardiovascular diseases and pancreatic cancer. This sensor uses a generic strategy involving in situ regeneration of directly electropolymerized molecular templates on laser‐engraved graphene for electroactive targets, and MIP films prepared on Prussian Blue (PB) layers for non‐electroactive molecules. The MIPs‐based sweat patch found an elevated leucine/BCAA ratio in sweat within an hour after BCAAs supplementation, with a good concentration correlation of leucine (R = 0.66) and BCAAs (R = 0.69) levels between serum and sweat. Additionally, healthy subjects experienced a higher increase in post‐ingestion leucine levels compared to other subgroups, suggesting that it could be an effective marker for metabolic syndrome monitoring.

#### 3.2.3 Wearable Sweat Sensors for Hormonal Detection

Hormones play a crucial role in regulating the physiological processes and behavior of the central nervous system. Notably, steroid hormone (e.g., cortisol) and neuropeptide Y (NPY) have emerged as potential biomarkers for monitoring stress‐related disorders (e.g., cardiovascular dysfunction) and mental health conditions (e.g., chronic stress and depression).^[^ ^174^ ^]^ Recent research has demonstrated a significant correlation between the concentrations of these hormones in sweat and blood, despite their markedly lower levels in sweat compared to serum. Although over 90% of endogenous cortisol in the bloodstream is bound to proteins, free cortisol can pass through the lipid bilayer membrane via passive intracellular transport and appear in sweat. Sweat‐based detection of stress‐related hormones can improve patient compliance,^[^ ^175^ ^]^ offering a more accessible and comfortable alternative to traditional blood sampling.

Monitoring stress indicators (e.g., cortisol or NPY) in sweat, has been extensively using antibodies,^[^ ^175^, ^176^, ^177^ ^]^ aptamers,^[^ ^36^, ^178^ ^]^ and MIPs.^[^ ^168^ ^]^ A recent study reported an immunosensing patch with laser‐engraved graphene electrodes,^[^ ^177^ ^]^ where cortisol in sweat competes with horseradish peroxidase (HRP)‐labeled cortisol for binding to immobilized antibodies. Quantitative detection is achieved by measuring the cathodic current generated by the HRP‐catalyzed reduction of hydrogen peroxide in the presence of a hydroquinone mediator. However, this design requires multiple preparation steps, including the addition of labels and washing, which could impede its practical adoption. In response, pseudoknot‐assisted aptamers and molecularly selective nanoporous membrane have been integrated into wearable sweat microfluidic devices,^[^ ^178^, ^179^ ^]^ providing efficient sample delivery and continuous cortisol quantification. For example, a newly identified cortisol aptamer was immobilized on semiconductor channels of a FET fabricated on a flexible PI substrate for on‐body cortisol detection (Figure 4e).^[^ ^36^ ^]^ In the presence of cortisol, the FET anchored aptamers undergo a reversible conformational change due to target binding‐induced rearrangement of backbone nucleotides. This modulates the surface charge and generates distinctive variations in the source‐drain current, enabling cortisol quantification for continuous stress management.

Other steroid hormones, such as oestradiol and testosterone, can also be present in sweat, and their personalized monitoring is of great interest in fertility and sexual health. A skin‐interfaced aptamer sensor based on target‐induced strand displacement (Figure 4f) has been reported for reagentless detection of the female hormone oestradiol in sweat.^[^ ^157^ ^]^ The sensing electrode consists of two interfaces for target recognition and signal transduction. The recognition interface, created by inkjet‐printing gold nanoparticles, is modified with anti‐oestradiol aptamers and partially hybridized ssDNA labelled with the electroactive MB (MB‐ssDNA). The other MXene‐modified electrode is functionalized with ssDNA complementary to the ssDNA‐MB for signal transduction. In the presence of oestradiol, the high affinity between the target molecule and the aptamer results in the release of the MB‐ssDNA, which is then recaptured by the MXene nanoelectrode, generating a quantitative SWV signal with a limit of detection of 0.14 pM. Additionally, an integrated microfluidic system, combining iontophoretic sweat stimulation and capillary burst valve guided sweat transport, is capable of monitoring periodic fluctuations of oestradiol in sweat during consecutive menstrual cycles, and has identified a high correlation with blood oestradiol levels.

#### 3.2.4 Wearable Sweat Sensors for Drug Monitoring

Although sweat is an unconventional source for monitoring drug metabolism compared to blood, urine, and ISF, its completely non‐invasive nature addresses compliance issues. Many therapeutic and illicit drugs in sweat, such as ethanol,^[^ ^180^, ^181^, ^182^ ^]^ nicotine,^[^ ^183^ ^]^ caffeine, and cathinone,^[^ ^184^ ^]^ correlate well with blood and plasma levels. In particular, sweat ethanol has been extensively explored using wearable sweat biosensors. For example, an integrated tattoo biosensor has been developed to measure sweat ethanol within 10 minutes using iontophoresis.^[^ ^182^ ^]^ Sweat ethanol provides a real‐time reflection of blood ethanol levels without the delays associated with transdermal devices and breathalyzers. The sensor achieves highly selective alcohol measurement by combining the enzymatic reaction of alcohol oxidase with the cathodic detection of peroxide products, and has been validated in healthy human subjects after the consumption of various levels of alcoholic beverages. It also features a Bluetooth interface for wireless signal transmission to mobile devices.

To date, monitoring of therapeutic drug levels in sweat has also been achieved for targets such as levodopa and psychoactive drugs. For example, a tyrosinase enzyme powered sweat band and a hydrogel‐based fingertip sweat sensor have demonstrated their functionality in guiding the systematic administering of levodopa.^[^ ^185^ ^]^ In addition, Zhang et al. developed a wearable electrochemical aptamer sensor array for quantifying multiple psychoactive drugs in sweat.^[^ ^184^ ^]^ This sensor combines dual aptamers with a multivariate discrimination method, allowing it to identify thirteen drugs and three interfering substrates with high sensitivity and specificity. However, to validate the usefulness and facilitate meaningful data interpretation, on‐body measurements and blood‐sweat correlation of these drugs still need to be verified.

Yet, to the best of the authors' knowledge, there are no reports of wearable sweat biosensors for antibiotics in the literature. One possible reason is that larger, hydrophilic antibiotic molecules, such as penicillin and amoxicillin, can be diluted significantly in sweat, imposing higher requirements on the detection limits of biosensors. Since sweat is a potentially useful medium for reflecting antibiotic levels (e.g., flucloxacillin, imipenem, and cefepime),^[^ ^186^ ^]^ this area remains ripe for further exploration to advance the field of non‐invasive therapeutic drug monitoring (TDM).

#### 3.2.5 Wearable Sweat Sensors for Macromolecular Detections

In sweat, proteins, particularly C‐reactive protein (CRP) and cytokines, are of great interest due to their roles in regulating inflammation and immune responses. These large, hydrophilic macromolecules enter sweat through paracellular diffusion and advective transport via intercellular canaliculi between adjacent cells, which results in significant dilution. Consequently, trace levels of sweat proteins are expected considering their ultra‐low concentrations in blood (e.g., picomolar to nanomolar). Nevertheless, their localized production in sweat is usually a result of systemic immune responses, suggesting that sweat cytokine levels are likely to reveal overall health status.

To date, several examples of wearable impedance sensors have demonstrated the potential to detect various inflammatory markers in natural sweat, including Interleukins,^[^ ^187^, ^188^, ^189^ ^]^ tumor necrosis factor‐alpha (TNF‐α),^[^ ^188^ ^]^ interferon‐gamma (IFN‐γ),^[^ ^190^ ^]^ and CRP. For example, a proof‐of‐concept clinical study was conducted on patients with inflammatory bowel disease (IBD) to measure CRP levels using a skin‐mounted microfluidic patch. By validating the analytical accuracy of the EIS antibody sensor against ELISA, CPR levels in sweat collected from 10 subjects were shown to be relatively independent of sweat rate, further supporting the feasibility of correlating sweat CRP levels with blood levels.

Recently, Tu et al.^[^ ^158^ ^]^ proposed an advanced wearable patch (Figure 4g) capable of wireless and continuous monitoring of CRP in patients with chronic obstructive pulmonary disease, heart failure, and infections. This patch combines iontophoretic sweat extraction, microfluidic sampling, and a graphene‐based sensor array, leveraging sweat flow to achieve fully automated CRP and antibody capture, followed by washing and electrochemical interrogation on the skin. The use of gold nanoparticle‐decorated graphene electrodes, along with a sensor array for pH, temperature, and ionic strength to calibrate for variations in sweat composition variations, achieved a detection limit for picomolar CRP levels. This method represents a generalizable approach for on‐body assessment of trace‐level biomarker dynamics, addressing unmet needs in personalized and non‐invasive protein biomarker monitoring.

Despite significant advances in sweat‐sensing prototypes for detecting various biomarkers, translation of these wearable sweat sensors into reliable robust proxies for blood measurements remains challenging. Like ISF, the partitioning of analytes in sweat differs from that in blood and is highly influenced by factors, such as size, charge state, hydrophobicity, and binding affinity to serum proteins. In addition, although sweat sampling is generally straightforward and minimally intrusive, the partitioning of analytes into sweat can vary based on different stimulation approaches and collection conditions. Sweat composition is also affected by sweat rate, which influences analyte dilution and ionic strength.^[^ ^105^ ^]^ These variations particularly complicate electrochemical sensing techniques that depend on stable electrical double layers and Debye length,^[^ ^191^ ^]^ such as EIS, FET, and SWV. Therefore, extensive research, especially large‐scale clinical studies, is needed to understand the relevance and effectiveness of sweat biomarkers and to fully realize their diagnostic potential.

## 4 Wearable Sensors for Wound Monitoring

**Figure 5:**
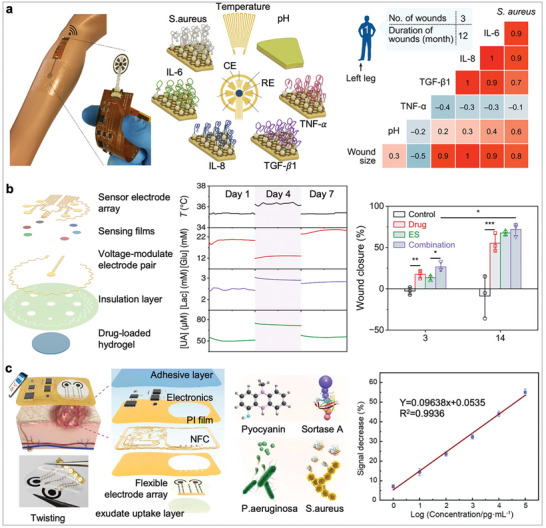
Wound exudate‐based wearable electrochemical biosensors. a) A soft, thin, multiplexed wound dressing for chronic wound monitoring. Left: Optical image of the monitoring patch on a wound area. Middle: a biomimetic passive microfluidic system for directional liquid transport. Right: Schematic of the multiplexing sensing array. Adapted under the terms of the CC‐BY license.^[^ ^34^ ^]^ Copyright 2021, The Authors. b) A multifunctional hydrogel‐based wearable wound patch for monitoring physiological conditions of the wound bed, as well as for antimicrobial treatment and electrically stimulated tissue regeneration. Reproduced under the terms of the CC‐BY license.^[^ ^195^ ^]^ Copyright 2021, The Authors. c) A MXene‐empowered battery‐free wound bandage for detecting bacterial virulence factors. The sensing electrode of the bandage is modified with AuNPs and MXene nanocomposite, enhancing sensitivity for multiplex biomarker profiling. Reproduced with permission.^[^ ^193^ ^]^ Copyright 2023, Elsevier.

Wound exudate is an epidermal fluid produced in response to tissue injury that provides a moist environment and contains the necessary nutrients for tissue repair. For example, metabolic byproducts in the exudate, such as glucose, lactate, uric acid, and ammonium, offer insights into the metabolic activity and bacterial presence at the wound site. High glucose levels can indicate bacterial infection, while elevated lactate and ammonium levels can suggest soft‐tissue hypoxia and angiogenesis in diabetic foot ulcers.^[^ ^192^ ^]^ Elevated levels of cytokines, including TNF‐α, interleukins (IL‐6, IL‐8), and transforming growth factor‐beta (TGF‐β1), indicate wound severity and excessive inflammatory responses. Therefore, comprehensive monitoring of these biomarkers in wound exudate provides substantial clinical insights into wound healing and helps identify potential complications (e.g., sepsis). Quantitative profiling of multiple biomarkers on the wound bed has been demonstrated with a multiplexed wound patch (**Figure** **5**a),^[^ ^34^ ^]^ which incorporates arrays of aptamer‐based electrodes. This ulcer care device can simultaneously detect four inflammatory mediators and one microbial burden, in addition to providing physiochemical information, such as temperature and pH. Of note, a nature‐inspired microfluidic collector, mimicking the skin structure of the Texas lizard, is employed to passively direct liquid flow, allowing for more efficient capture of wound fluid and delivery to the sensing areas.

Additionally, detecting specific microorganisms is also crucial for understanding the microbial burden and infection status of the wound.^[^ ^193^ ^]^ For example, Xiong et al. introduced a wearable wound patch to monitor the dynamics of opportunistic pathogen growth.^[^ ^194^ ^]^ By utilising a DNA crosslinked hydrogel that responds to deoxyribonuclease (DNase) enzyme, the sensor detects changes in the dielectric properties of the hydrogel‐coated electrode when exposed to pathogenic bacteria, including *Staphylococcus aureus*, *Pseudomonas aeruginosa*, and *Staphylococcus pyogenes*. This enables early detection and timely intervention of wound infections before they become visibly apparent. Moreover, the wound patch eliminates the need for an internal power source by employing near‐field communication (NFC) technology for wireless power transmission, which significantly enhances its practicality and usability in real‐world healthcare settings.

Another remarkable innovation is a therapeutic wound patch that combines monitoring and treatment functions (Figure 5b).^[^ ^195^ ^]^ Constructed from a biocompatible, highly elastic, and stretchable thermoplastic substrate, this wearable patch adheres conformally to the wound bed, reducing skin irritation and undesirable deformation. In addition to analyzing essential wound biomarkers and exogenous factors (e.g., bacteria), the wound patch features voltage‐modulated electrodes designed induce drug and electrical stimulation to enhance wound healing. Under an external electrical potential, an electro‐responsive hydrogel, composed of chondroitin 4‐sulfate and glucosamine, releases antimicrobial peptide (AMP) on‐demand in response to electrical stimulation. Compared to the control group, this combined therapy promotes cell proliferation, motility, tissue regeneration, and more effective pathogen elimination, resulting in a higher rate of wound closure. Demonstrated in a preclinical splinted excisional wound model in diabetic rodents, this approach represents a versatile and efficient method for treating chronic cutaneous wounds.

Despite these advancements, several challenges remain for wearable wound care platforms. One major challenge is the heterogeneous composition of biomolecules in wound fluid, including high levels of protein, migrated cells and bacteria. This complexity can lead to electrode biofouling and hinder accurate measurements over prolonged use. This issue can be mitigated by adding a membrane layer to limit the diffusion of interferents and protect the electrode,^[^ ^195^ ^]^ as well as by functionalizing electrodes with nanomaterials (e.g., MXene, Figure 5c).^[^ ^193^ ^]^ Additionally, the absence of a continuous wound fluid sampling and circulation system affects data accuracy, as the mixing of newly secreted wound exudates can delay response and compromise temporal resolution.^[^ ^156^ ^]^ Further in‐depth studies are needed, including investigations into the cellular and molecular mechanisms underlying wound healing facilitated by the patch, and preclinical assessments using pig models with anatomical similarities to human skin, to advance the clinical application of wearable wound biosensors.^[^ ^195^ ^]^

## 5 Wearable Sensors for Oral‐Cavity Monitoring

**Figure 6:**
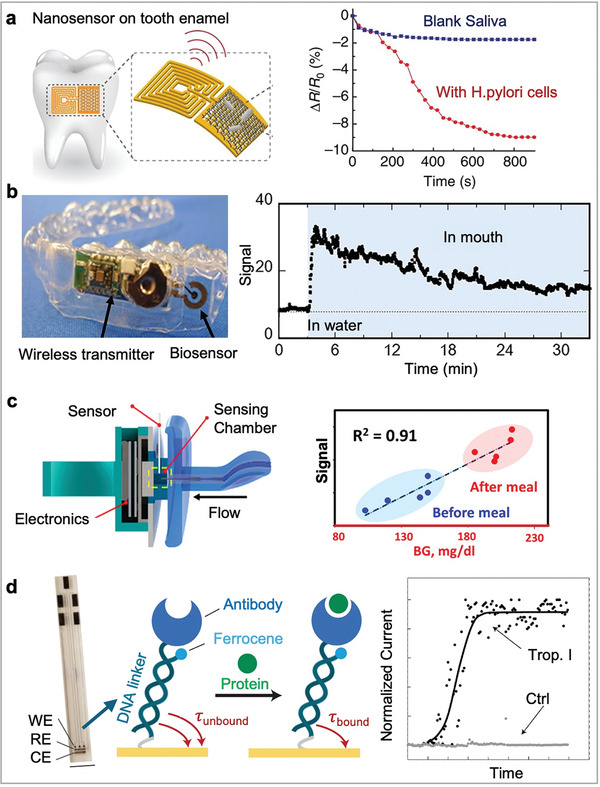
Saliva‐based wearable electrochemical biosensors. a) A wearable graphene‐based nanosensor for detecting bacteria on tooth enamel, which is achieved through the specific binding of pathogenic bacteria with graphene anchored peptides. Reproduced with permission.^[^ ^30^ ^]^ Copyright 2012, Springer Nature. b) A mouthguard‐based wearable device for enzymatic biosensing of salivary glucose. Reproduced with permission.^[^ ^197^ ^]^ Copyright 2020, American Chemical Society. c) A baby‐friendly pacifier for glucose biosensing. Reproduced with permission.^[^ ^200^ ^]^ Copyright 2019, American Chemical Society. d) Modulation of molecular pendulum dynamics upon protein binding. The MP based biosensor achieved continuous monitoring of salivary cardiac troponin I in a living rodent model. Reproduced with permission.^[^ ^214^ ^]^ Copyright 2021, Springer Nature.

Saliva, an easily obtainable diagnostic fluid with plethora biomolecular information, has garnered significant attention for monitoring of human health. Several metabolites, hormones, proteins, and exogenous substrates (e.g., drugs and microorganisms) can be found in saliva, entering from the blood via transcellular or paracellular pathways, and serving as effective indicators of the body's physiological state. Additionally, the relatively large volume of saliva, secreted mainly by the parotid glands, facilitates the use of in‐month sensing wearables without the need for complicated sample processing and collection.^[^ ^196^ ^]^ These attractive features drive the expanding applications of saliva analysis. The first wearable oral biosensor was introduced in 2012 by Mannoor et al., designed as a tooth cavity modifier for monitoring bacterial film development (**Figure** **6**a).^[^ ^30^ ^]^ This oral‐cavity sensor was fabricated by printing graphene onto water‐soluble silk and transferring it directly onto the tooth enamel. To recognize bacteria, a naturally occurring microbial‐specific peptide was immobilized on the graphene, and an electrical impedance instrument was used for signal transduction. This sensor enables the detection of salivary bacteria at the single‐cell level in vitro, and features a resonant coil electrode for wireless, battery‐free microorganism detection.

Building on previous in vitro studies that showed good correlations between blood and saliva metabolite levels, several modern oral‐cavity wearable biosensors have been developed, particularly using mouthguard platforms (Figure 6b),^[^ ^197^ ^]^ for continuous tracking of salivary metabolites. For example, Wang's group developed a lactate monitoring system via the integration of a screen‐printed, LOx enzyme loaded electrode with a mouthguard device.^[^ ^198^ ^]^ To resist fouling from saliva, a poly‐o‐phenylenediamine coating was electropolymerized on the electrode surface, enhancing sensing stability for over 2 h. The same group expanded this wearable concept to detect uric acid and glucose in saliva as proxies for blood levels.^[^ ^33^, ^91^ ^]^ Additionally, alternative configurations for oral biosensing, including a custom‐fitted and miniaturized mouthguard,^[^ ^199^ ^]^ and a baby‐friendly pacifier (Figure 6c),^[^ ^200^ ^]^ have been introduced for measuring salivary glucose within physiologically relevant range. Since the rigid mouthguard may be prone to causing injuries and may not be suitable for newborns due to their sensitive skin, a novel pacifier‐based biosensing prototype was designed specifically for infants. This design places the sensing chamber outside the oral cavity, eliminating the risk of transducer material leakage into the mouth. It also features a nontoxic polymeric nipple with a safety rectifying channel made from silicone with asymmetrical conical constrictions. This ensures efficient, unidirectional saliva flow from the infant's mouth to the electrochemical chamber, thus enabling safe and effective saliva collection without backflow.

In addition to enzyme‐catalyzed analytes, a diverse matrix of constituents in saliva has been detected using biorecognition elements, including small molecules (e.g., adenosine monophosphate),^[^ ^201^ ^]^ hormones (e.g., cortisol),^[^ ^36^, ^202^, ^203^ ^]^ stress‐related enzymes (e.g., α‐amylase),^[^ ^204^ ^]^ nucleic acids (e.g., SARS‐CoV‐2 RNA),^[^ ^205^ ^]^ cytokines (e.g., immunoglobulin A,^[^ ^206^ ^]^ IL‐6 and IL‐8),^[^ ^207^ ^]^ microorganisms(e.g., Zika Virus,^[^ ^208^ ^]^ foot‐mouth disease virus,^[^ ^209^ ^]^ oral bacteria),^[^ ^210^, ^211^ ^]^ and drugs (e.g., ampicillin,^[^ ^212^ ^]^ chemotherapeutic agent cisplatin,^[^ ^213^ ^]^ cotinine).^[^ ^74^ ^]^ Notably, Kelley's group reported an antibody‐based molecular pendulum (MP) for reagent‐free electrochemical biosensing of protein biomarkers associated with heart disease (i.e., troponin I).^[^ ^214^ ^]^ This novel sensing scheme utilized the electrical field‐mediated motion of an inverted molecular pendulum (Figure 6d), which is constructed using double‐stranded DNA as a linker, a specific antibody for target recognition, and a redox reporter for signal transduction. By quantifying the electron transfer kinetics modulated by analyte binding, the MP sensor can detect cardiac troponin with a remarkable limit of detection of 40 fM. For in vivo applications, the authors demonstrated its potential for tracking protein dynamics in the saliva of live mice, which is treated with troponin or cardiotoxic doxorubicin to induce troponin release in the saliva.

Although many of these findings have been validated using real human saliva, integrating these analyses with wearable saliva devices for in situ oral monitoring has progressed slowly. Two primary challenges hinder this progress. First, saliva contains a substantial number of proteins, food debris, and potential contaminants from gum bleeding, which exacerbate electrode fouling. Second, the dilution of biomarkers in saliva, as along with variations in viscosity, composition, and secretion rate, pose significant challenges to analytical accuracy and stability. For example, in diabetes, variations in neural and hormonal balance can influence the glucose transport from the blood to the salivary glands, therefore affecting measured salivary glucose levels.^[^ ^215^ ^]^ Nevertheless, the development of novel bioreceptors, particularly in connection with wearable oral device, is expected to advance the diagnostic scope of saliva, especially for on‐body management of chronic diseases and drug abuse.

## 6 Wearable Sensors for Ocular Monitoring

The interest in tears as a diagnostic fluid has also emerged rapidly with advances in wearable biosensing electronics. Tears, which are secreted by the lacrimal glands to act as a protective film layer covering the eyeball, contain many important biomarkers (e.g., metabolites, hormone, proteins, antibodies) that diffuse directly from the bloodstream.^[^ ^216^, ^217^ ^]^ Compared to blood, tears are less susceptible to fouling due to the natural antifouling mechanisms of the eyeball. Despite these advantageous characteristics, sampling tears for in vitro diagnosis remains challenging due to their relatively small volume, ease of evaporation, and variations in composition and secretion rate. For instance, different in vitro tear collection strategies, such as Schirmer's strip or glass capillary tubes, can affect the concentrations of sampled analytes.^[^ ^215^ ^]^ In contrast, contact lens‐based wearable devices are popular and attractive for tear sensing, because they can be worn with minimal irritation.

**Figure 7:**
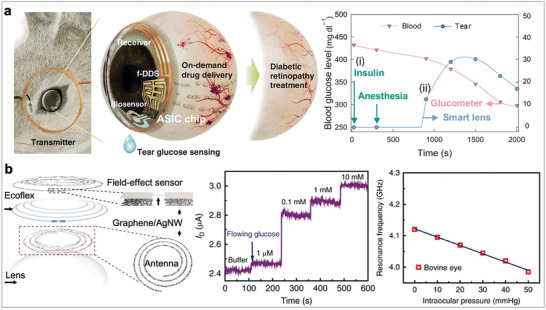
Tear‐based wearable electrochemical biosensors. a) A wireless smart contact lens for diabetic diagnosis and therapy. Reproduced with permission.^[^ ^219^ ^]^ Copyright 2020, American Association for the Advancement of Science. b) A stretchable and transparent contact lens utilizing a graphene and silver nanowire‐based FET sensing strategy for real‐time glucose detection and wireless monitoring of intraocular pressure. Reproduced under the terms of the CC‐BY license.^[^ ^220^ ^]^ Copyright 2017, the Authors.

Similar to other biofluids, tears, especially basal tears produced without external stimuli or emotions, contain glucose that correlate well with blood glucose levels. In 2012, Parviz's team made early advancements in this field by equipping a contact lens with GOx for glucose monitoring in tears. They implemented a dual‐electrode configuration to address interference issues, and embedded wireless read‐out and power systems to facilitate accurate, long‐term glucose sensing in tears.^[^ ^218^ ^]^ With the development of soft materials and fabrication that offer high miniaturization capabilities, efforts have also focused on the combination of diagnostic and drug delivery functions. For example, Hahn's group reported a multifunctional smart contact lens for monitoring and treating diabetic retinopathy (**Figure** **7**a).^[^ ^219^ ^]^ This device integrates biosensing, data processing, a voltage‐controlled drug releasing system (f‐DDS), and a resonant inductive unit coupled with a copper coil for wireless power transfer. It demonstrated real‐time ocular glucose biosensing using a rabbit model, and importantly, the safe and on‐demand medication of genistein for treating diabetic retinopathy.

Other physiological signals, such as intraocular pressure,^[^ ^220^ ^]^ stress levels and chronic ocular disease status,^[^ ^221^, ^222^ ^]^ have also been monitored by tear‐based wearables. For example, a stretchable contact lens (Figure 7b) achieved real‐time monitoring of glucose level and intraocular pressure of eyeballs, by integrating resistance, inductance and capacitance components into an electronic resonant circuit. Another notable antibody‐based contact lens was developed for detecting matrix metalloproteinase‐9 (MMP‐9), an essential endopeptidase indicator for chronic ocular surface inflammation (OSI). The gFET‐based sensing electrode, functionalized with an antibody specific to MMP‐9, enables continuous and quantitative analysis of this OSI indicator from 2 to 500 ng mL^−1^. Additionally, a skin‐attached heat patch, designed to warm the eyelids to relieve OSI pathologies, was incorporated for therapeutic purposes and communicates wirelessly with the diagnostic compartment via a smartphone.

Recent advancements have also shown the potential for quantifying other ocular protein levels in human tears (e.g., TNF‐α,^[^ ^223^ ^]^ vascular endothelial growth factor) using aptamer and MIP based bioreceptors.^[^ ^224^ ^]^ For example, Wang et al. designed an aptamer‐based gFET nanosensor to determine TNF‐α concentration in tear diagnostics.^[^ ^223^ ^]^ The immobilization of a PEG monolayer with optimized molecular weight on the surface of graphene FET reduced nonspecific absorption of background interferents, such as IFN‐γ and human interleukin‐002, thereby allowing for more sensitive and reproducible analytical performance. With the continued development of new bioreceptors, tear‐based wearable sensors could expand to detect additional proteins, with albumin, lactoferrin and lysozyme being among the most abundant. However, as with other non‐blood biofluids, extensively evaluation of device performance on animal subjects or human wearers, as well as the specificity of biomarkers during ocular disease progression, is required.

In the commercialization process of contact lens biosensing platforms, a notable acceleration occurred with Google's partnership with Novartis.^[^ ^31^ ^]^ Combining expertise in electronics miniaturization and medical device development, they created a prototype electrochemical enzymatic biosensor. This device, consisting of a miniaturized electrochemical transducer and antenna embedded within a hydrogel matrix, provides real‐time glucose concentrations in surrounding tears. Additionally, a spring‐like glucose biosensor composed of coiled wire electrodes, was designed to be placed behind the lower eyelid for constant access to tear fluids.^[^ ^225^ ^]^ This prototype was coated with a polysaccharide hydrogel to enhance comfortability and eliminate painfulness. However, similar to Google one, the subsequent release of this product has been delayed, highlighting the technological challenges of achieving high performance wearable tear glucose sensors, such as inconsistencies between tear and blood glucose levels and complexities of the on‐eye environment.

## 7 Recent Advances in Wearable Electrochemical Biosensors

Recent groundbreaking innovations in wearable electrochemical biosensors have significantly expanded the range of detectable targets, and enhanced the analytical performances, functionality, and operational longevity of these systems.

### 7.1 State‐of‐the‐Art Electrochemical Techniques

While electrochemical electronics have a smaller footprint compared to other wearable biosensing platforms (e.g., fluorescence and plasmonic methods), a critical drawback for traditional electrochemical methods is their insufficient sensitivity, which makes it difficult to distinguish the dynamics of biomarkers at clinically relevant levels. Beyond traditional electrochemical sensors, OECTs have emerged as a promising technology for wearable biosensing applications due to their capability to improve sensing quality through the synergy of electrochemistry and signal amplification.^[^ ^226^, ^227^, ^228^ ^]^ Specifically, OECTs operate based on the reversible electrochemical doping process of the organic mixed ion‐electron semiconducting channel, which allows for significant signal amplification due to full ionic penetration from the electrolyte.^[^ ^226^, ^229^, ^230^, ^231^ ^]^ This unique modulation mechanism enhances detection accuracy, making OECTs particularly useful for detecting relatively weak signals in complex biofluids and for analyzing lowly abundant macromolecules. Moreover, OECTs offer other distinctive advantages, such as compact size, low power consumption, and tuneable sensitivity and detection ranges by adjusting the gate voltage, which is ideal for on‐body measurements.

**Figure 8:**
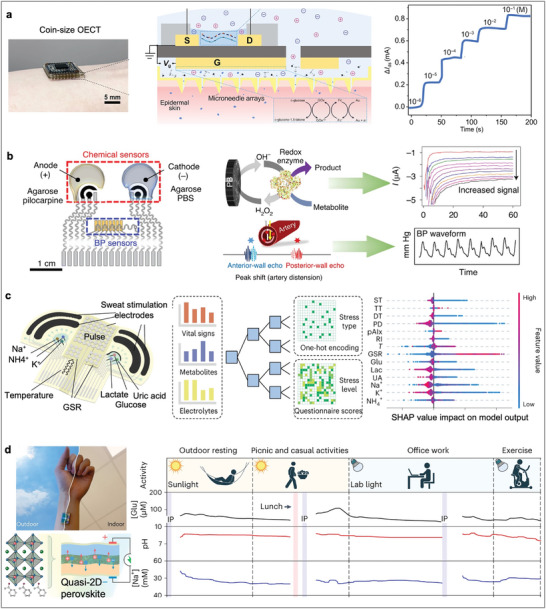
Recent advances in wearable electrochemical biosensors. a) Optical image, sensing mechanism of a fully integrated OECT‐CGM prototype, and its signal response to different glucose levels. Reproduced under the terms of the CC‐BY license.^[^ ^38^ ^]^ Copyright 2024, the Authors. b) A wearable patch combining acoustic and electrochemical sensors for simultaneously tracking haemodynamic parameters and molecular concentrations in ISF and sweat. Reproduced with permission.^[^ ^35^ ^]^ Copyright 2021, Springer Nature. c) A wearable electronic skin for monitoring multiple physiochemical parameters to track stress response. It is empowered by machine learning pipeline for stressor classification and stress level assessment. Shapley additive explanations (SHAP) plot for evaluation of state anxiety level based on the dataset collected by the wearable stress monitor is also represented. Reproduced with permission.^[^ ^234^ ^]^ Copyright 2024, Springer Nature. d) A perovskite solar cell powered wearable sweat biosensor. This device enables all‐day monitoring of physicochemical parameters under different light conditions. Reproduced with permission.^[^ ^235^ ^]^ Copyright 2023, Springer Nature.

For example, a fully integrated OECTs‐based MN sensing array (**Figure** **8**a) was developed for subcutaneous glucose measurement, demonstrating enhanced reliability and resistance to noise. During in vivo validation, the OECTs‐empowered glucose sensor exhibited a high current response and a comparably high signal‐to‐noise ratio (SNR), even with minor glucose fluctuations, underscoring its sensitivity and resilience in noisy biofluidic environments.^[^ ^38^ ^]^ Beyond CGM, similar advancements using OECTs have been achieved for other on‐body applications, including antibody‐based measurement of sweat cortisol,^[^ ^232^ ^]^ and aptamer‐based sensing of transforming growth factor and antibiotic tobramycin.^[^ ^226^, ^233^ ^]^

### 7.2 Closed‐Loop Control

There is growing interest in the “sense‐and‐act” system that enableon‐demand delivery of therapeutic drugs based on biomarker feedback,^[^ ^236^, ^237^ ^]^ facilitating tailored drug dosages and precise medicine. For example, a closed‐loop controlled sweat patch was developed for transdermal diabetic detection and therapy.^[^ ^238^ ^]^ This system features a closed‐loop transdermal therapy module in which a hyaluronic acid hydrogel matrix is embedded with temperature‐responsive nanoparticles loaded with an antidiabetic drug (i.e., metformin). The multiple sweat‐uptake layers allow effective sweat collection and reliable analysis, even with a sample volume of approximately 1 µL. Moreover, the use of porous gold nanostructures to maximize the electrochemically active electrode surface, along with glutaraldehyde cross‐linked GOx enzyme, ensures the robustness and reliability of the sweat sensor under mechanical friction and deformation. Based on measured sweat glucose levels self‐calibrated according to pH, temperature and humidity parameters, the sweat patch demonstrated on‐demand drug release in a type‐2 diabetic mouse model. A similar strategy has also been demonstrated in a transdermal MN patch, combining hollow MNs for ISF glucose sensing with an electrically controlled electroosmotic pump for insulin delivery. In vivo validation of this prototyping device was demonstrated in a rodent model by successfully stabilizing glucose levels within a normal and safe range.^[^ ^236^ ^]^

### 7.3 Muti‐Sensory Integration

A notable breakthrough in biosensing involves the development of muti‐sensory wearable systems that combine several biosensing components with physical sensing modalities (Figure 8b). These systems can simultaneously track the dynamics of chemical biomarkers (e.g., glucose from ISF, lactate caffeine, and alcohol from sweat) and vital physical parameters (e.g., blood pressure and heart rate).^[^ ^35^ ^]^ The integration of these components was achieved through rational design approaches, including the use of stretchable styrene‐ethylene‐butylene‐styrene block copolymer‐based materials, optimised modality layouts, and solvent‐soldering fabrication processes. This hybrid patch represents a considerable advancement in providing comprehensive and continuous healthcare information, and alerting wearers to a variety of abnormal physiological changes. Additionally, the fusion of biosensing functionalities allows for the collection of biochemical information and physiological signals using a sweat sensing patch. Specifically, a wearable hybrid electronic skin (Figure 8c) was developed to assess stress indictors,^[^ ^234^ ^]^ including three vital electrophysiological signs (pulse waveform, galvanic response and skin temperature), and six molecular substrates in sweat. The comprehensive physiological information provided by the wearable patch enables the classification of different stressors (e.g., cold pressor, virtual reality challenge, and intense exercise) and the use of machine learning (ML) for predicting states anxiety.

### 7.4 Power Supply

Exploring self‐powered and sustainable energy sources (e.g., solar,^[^ ^235^ ^]^ biochemical energy, and human motion) for wearable electronics offers a convenient and potentially safer alternative to lithium batteries, which face limitations such as bulkiness, limited lifespan, and safety hazards.^[^ ^239^ ^]^ Self‐powered wearables have been successfully developed to harvest energy from body movements, biofluids, and external sources. For example, biofuel cells utilize biochemical energy generated from enzymatic reactions involving lactate or glucose in biofluids.^[^ ^240^ ^]^ In addition, triboelectric and piezoelectric nanogenerators harness mechanical and kinetic energy from body movements to generate electricity, providing a continuous power supply without the need for external charging.^[^ ^241^, ^242^ ^]^

A notable demonstration of utilizing solar energy to power wearable biosensing electronics was reported by Gao's Group, which developed a flexible perovskite solar cell (Figure 8d).^[^ ^235^ ^]^ This cell converts ambient light into power for continuous metabolic monitoring in sweat. Due to its favourable intrinsic features (e.g., high absorption coefficients, tuneable bandgap, and flexibility), the quasi‐2D perovskite crystal powered photovoltaic cells can be tailored to various lighting conditions, including natural sunlight and artificial indoor lighting. This enables a sufficient and steady power supply for wearables, allowing light‐powered sensing platform to monitor multimodal phytochemical data for over 12 h without the need of battery recharging or vigorous exercise.

## 8 Challenges and Future Perspectives

**Figure 9:**
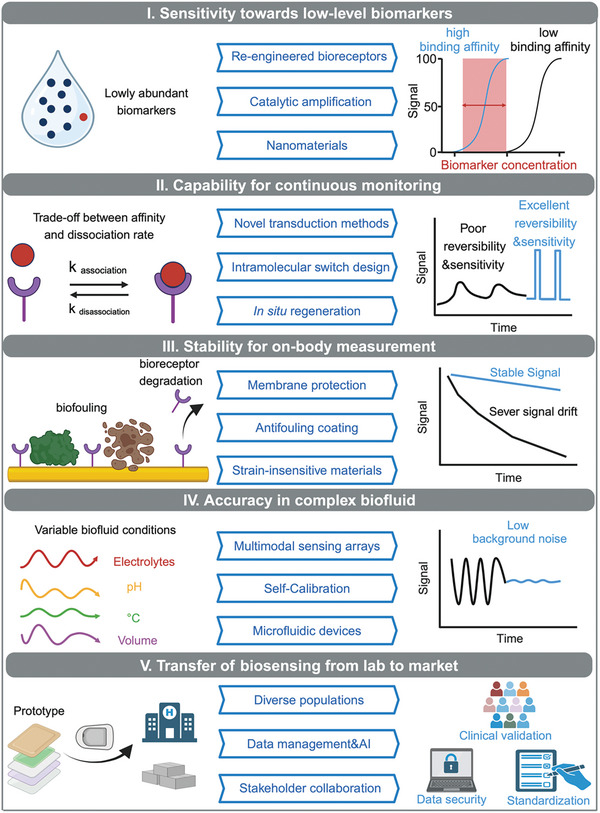
Challenges and future perspectives in developing wearable electrochemical biosensors.

In this review, we provided a state‐of‐the‐art overview of emerging wearable electrochemical biosensing platforms for biomedical applications. Significant advances have been made in wearable electrochemical biosensors, enabling real‐time and continuous biomarker profiling in various biological fluids, including ISF, sweat, wound exudate, saliva, and tear. The development and adoption of biosensors for a wide range of applications, particularly those related to metabolic syndromes, mental health, chronic diseases, and the monitoring of therapeutic drugs, are among the most emerging technological goals. To this end, the wearable electrochemical biosensing devices have been designed and tailored, so that parameters, such as analytical performance, functionality and flexibility, meet the requirements for specific body regions. Yet, several challenges (**Figure** **9**) remain in realizing the full potential of wearable sensing devices, such as long‐term robustness, in vivo accuracy, and regulatory frameworks for data security and commercialization. These issues need to be addressed to facilitate commercialization and widespread adoption in clinical practice.

### 8.1 Sensitivity toward Low‐Level Biomarkers

Detecting analytes at low concentrations such as nucleic acids, proteins, or cells, poses a significant challenge for on‐body measurement as these biomarkers often exist at femtomolar to picomolar levels, or even lower. To improve sensitivity and limit of detection, several strategies have been reported for electrochemical biosensors, including re‐engineering of recognition elements,^[^ ^243^, ^244^ ^]^ enzyme‐catalyzed amplification,^[^ ^245^ ^]^ the use of nanomaterials (e.g., gold nanostructures and 2D nanomaterials),^[^ ^75^, ^246^, ^247^ ^]^ and novel sensing mechanisms (e.g., pendulum molecular switches).^[^ ^214^, ^248^, ^249^, ^250^ ^]^ These amplification strategies can enhance sensitivity by several orders of magnitude. For example, nanomaterials, with their large surface area‐to‐volume ratio and excellent electrochemical properties, can significantly increase the number of immobilized biorecognition elements and enhance charge/electron transfer efficiency. However, the high surface area also increases the risk of nonspecific adsorption and potential biocompatibility concerns, which need to be carefully evaluated for real‐word applications. In addition, biomolecular nanotechnology, such as DNA nanostructures and nano‐enzymes,^[^ ^249^ ^]^ can improve analyte association through enhanced surface probe distribution and target interaction.

### 8.2 Capability for Continuous Monitoring

Continuous biosensors offer a revolutionary approach to healthcare, by relying on reversible or rapidly regenerative interactions between targets and biorecognition elements. However, there is a trade‐off between receptor sensitivity and their reversibility.^[^ ^251^ ^]^ Bioreceptors suitable for continuous monitoring need high binding/dissociation rates for rapid equilibration with the surrounding fluid, enabling near real‐time response to concentration variations. This high temporal resolution often comes at the cost of impaired detection limits and is more suited for highly abundant substances (i.e., millimolar levels).^[^ ^252^ ^]^ Most biomarkers in the human body are at picomolar to femtomolar levels, making continuous monitoring challenging. A feasible strategy to address this as we described above is the adoption of highly sensitive electrochemical transduction methods (e.g., FET or OECT). In addition, bioreceptor modification, such as nucleotide substitutions and allosteric inhibition,^[^ ^253^ ^]^ can be applied to alter the detection limits while maintaining dissociation kinetics. Similarly, intramolecular strand‐displacement switch designs can improve disassociation kinetics for bioreceptors without compromising detection limits.^[^ ^254^ ^]^ Another feasible strategy involves the regeneration of bioreceptors through chemical reagents or electrochemical regeneration to restore bioreceptors,^[^ ^156^, ^157^, ^158^, ^255^ ^]^ for which microfluidic systems for precise in situ liquid handling can be adopted.^[^ ^157^, ^158^ ^]^

### 8.3 Stability for on‐Body Measurement

Biofouling of sensing electrodes is a unique yet critical issue for electrochemical biosensors deployed in complex matrices. This problem not only impairs sensitivity and leads to false results, but also reduces the longevity of the wearable sensing device due to signal drift and degradation.^[^ ^256^, ^257^ ^]^ To combat this issue, different antifouling strategies have been explored, including self‐assembled monolayers,^[^ ^258^ ^]^ antifouling coatings,^[^ ^259^ ^]^ and membrane protection.^[^ ^260^, ^261^ ^]^ These strategies help electrodes and biorecognition elements withstand harsh biological environments against hydrolysis, oxidation, and endonuclease activity. Additionally, the durability of wearable and implantable materials is crucial for maintaining sensor functionality during continuous motion and external pressure. Innovations in flexible and stretchable materials that conform to the human body and retain their mechanical and electrical properties are essential for this purpose.^[^ ^262^ ^]^

### 8.4 Accuracy in Complex Biofluids

Biological fluids exhibit significant compositional variability due to diet, hydration levels, circadian rhythms, and individual physiological differences. For example, the epidermal environment is highly variable in terms of skin temperature, humidity, and pH. These fluctuations can affect enzyme reactivity, bioreceptor binding affinity and the electrochemical interface. To mitigate these influences, multimodal sensing arrays can be constructed to simultaneously collect of multivariate information (e.g., temperature, pH, ionic concentrations) for self‐calibration, therefore enhancing accuracy. Moreover, sample contaminants and evaporation, particularly in sweat, wound exudate, saliva and tears, can further alter analyte concentrations complicating data interpretation and reducing the accuracy of on‐body measurements. Therefore, wearable biosensors must be designed to minimize background noise from non‐target interferents and environmental variations. In addition to adopting previously described protective layers (e.g., membrane coatings) to repeal containments,^[^ ^263^ ^]^ sensors with fast sampling and rapid turnaround time can minimize evaporation effects by timely processing and analyzing sample fluids. Microfluidic channels can be designed to isolate and transport fresh samples, avoiding the mixture of new and old biofluids and eliminating contamination.^[^ ^21^, ^86^ ^]^

### 8.5 Transfer of Biosensing from Lab to Market

Extensive clinical validation of wearable biosensors is essential for regulatory approval and widespread acceptance in the healthcare industry.^[^ ^264^ ^]^ Clinical trials should assess sensors for biocompatibility and safety, ensuring minimal component toxicity and immunogenicity,^[^ ^265^ ^]^ and low irritation and stimuli in diverse populations and conditions.^[^ ^266^, ^267^ ^]^ Long‐term studies following patients over extended periods are valuable for understanding biosensor performance and outcomes in healthcare surveillance. In addition, with the capabilities of continuous and multiplexed monitoring, wearable biosensors generate vast amounts of data, which pose risks on data breaches. It is essential to develop high‐security systems to resist cyber‐attacks or hacking incidents. This requires advanced encryption techniques, secure data transmission protocols, and stringent access controls to protect the sensitive health information of users. Machine learning algorithms are powerful digital tools for interpreting complex datasets and uncovering correlations between analyte profiles and health conditions.^[^ ^268^ ^]^ Data fusion from multimodal sensing modalities can provide a comprehensive health profile, leading to more accurate predictions and early warning of potential health issues. Moreover, developing standardized protocols for sensor interrogation, sample collection, device fabrication and data interpretation is also essential for consistency and reliability. These protocols should cover all aspects of sensor use, from initial setup and calibration to data analysis and reporting.^[^ ^269^ ^]^ Close collaboration with international standards organizations, regulatory bodies, healthcare providers and industry stakeholders are required to establish these standards. Ensuring compliance with these standards is critical for gaining regulatory approval and achieving widespread clinical application of wearable sensors. By addressing these obstacles, the widespread acceptance and use of wearable electrochemical biosensors for advanced healthcare monitoring is achievable.

## Conflict of Interest

The authors declare no conflict of interest.
